# Voluminous hiatal hernias – the role of robotic surgery

**DOI:** 10.1515/iss-2023-0033

**Published:** 2024-07-26

**Authors:** Michel Dosch, Mickael Chevallay, Minoa K. Jung, Stefan Mönig

**Affiliations:** Surgery Department, The Division of Digestive Surgery, University Hospitals of Geneva, Geneva, Switzerland

**Keywords:** giant or voluminous hiatal hernia, hiatal hernia repair, minimal invasive surgery, robotic surgery, laparoscopic surgery, Toupet fundoplication

## Abstract

Robotic surgery has become increasingly prevalent in UGI surgery over the last decade, particularly for treating hiatal hernias. Voluminous hiatal hernias, defined as the herniation of 30–50 % of the stomach into the thorax, often require surgical intervention due to associated dysphagia and potential severe complications. Given the challenges of repairing voluminous hiatal hernias, especially in elderly and fragile patients, the surgical technique should be optimal. Robotic surgery affords excellent visualization, allowing high mediastinal dissection and precise hiatus reconstruction. Despite the clear technical advantages, it remains to be demonstrated if the robotic approach matches the outcomes of conventional laparoscopic techniques. We review here the fundamentals of hiatal hernia surgery and describe our surgical technique using the da Vinci Xi robot to operate voluminous hiatal hernias. Additionally, we performed a systematic research analysis and selected recent publications focusing on robotic surgery for voluminous hiatal hernias. Recent studies report comparable complication rates, recurrence, and hospital stay lengths between robotic and laparoscopy surgery. Initial robotic procedures had longer operative times, which decreased with surgeon experience. Most of the studies were observational and retrospective, reporting the experience of a single center. Robotic surgery appears to be a viable option with similar complications rates to laparoscopic surgery under optimized conditions. Current literature supports the broader adoption of robotic surgery for voluminous hiatal hernias. However, prospective randomized studies are needed to further validate its use.

## Introduction

A hiatal hernia is defined as an abnormal protrusion of components of intra-abdominal content through the esophageal hiatus. Hiatal hernia is the most common form of internal hernia. The likelihood of a hiatal hernia increases with age. Obesity is considered the main risk factor and men are affected more than women [1].

A distinction is made between axial and paraesophageal hiatal hernia. We historically distinguish three types of hiatal hernias, including sliding hernias (type 1), which are the most prevalent form; paraesophageal hernias (type 2); and hernias not included in types 1 and 2 (type 3) [2]. Voluminous hiatal hernias are usually defined as the migration of more than 30–50 % of the stomach into the thorax [3]. This condition is relatively rare, accounting for less than 5 % of hiatal hernias. However, a precise estimate of the incidence or prevalence of voluminous hiatal hernias is limited due to a lack of consensus on their definition. It’s important to note that an aging population is expected to lead to an increased prevalence of hiatal hernias, particularly voluminous ones, requiring more complex operations in potentially more fragile patients [4].

The etiology of voluminous hiatal hernias is still not entirely clear. Different mechanisms have been proposed including increased intra-abdominal pressure (obesity, pregnancy) or decreased thoracic pressure (chronic lung disease) forcing the gastroesophageal junction into the thorax [5], 6]. Shortening of the esophagus due to fibrosis (severe chronic GERD, excessive vagal nerve stimulation) can also contribute to the development of this type of hernia [7]. Voluminous hiatal hernias may also be observed after surgery involving opening of esophageal hiatus such as esophagectomy, antireflux surgery, and hiatal hernia repair.

According to the German S2k-guidelines for GERD published in 2022 and American SAGES guidelines published in 2013, the indication for surgical repair of paraesophageal hiatal hernias is given for symptomatic patients [8], 9]. For asymptomatic patients, the decision depends on other factors including age and risk of complications. Indication for surgery is always given in the case of a voluminous hiatal hernia due to the high risks associated with nonoperative management, such as torsion, perforation, hemorrhage, and gangrene, which can increase mortality [2], 10]. While laparoscopy is considered superior to laparotomy for hiatal hernia surgery, the role of robotic surgery remains a matter of debate and has yet to be demonstrated in prospective multicenter randomized control trials. Here, we discuss the role of robotic surgery in the treatment of voluminous symptomatic hiatal hernias.

## Methods

In order to investigate the benefit of robotic surgery for hiatal hernias repair, we conducted a literature research on PubMed using the following keywords: minimal invasive surgery, robotic surgery, and hiatal hernia. Only articles written in English were considered for primary review. We included only studies focusing on robotic surgery and excluded articles focusing on other methods.

## Diagnostic

Hiatal hernias do not necessarily have to cause symptoms. Often a hiatal hernia remains unnoticed and is discovered incidentally. Symptomatic disease may manifest as gastroesophageal reflux disease (GERD), obstruction, or upper gastrointestinal bleeding. Hiatal hernias should be treated according to the symptoms caused.

In axial hernias, the upper part of the stomach slides through the esophageal hiatus. As this can irritate and affect the opening of the esophagus, axial hernia often causes GERD, which is usually treated with medication initially [11]. According to the guidelines, surgical treatment is indicated when GERD symptoms are persistent despite appropriate medical therapy or when the patient does not wish to take medication lifelong, which may be particularly important in younger patients [8]. The best surgical results are achieved in patients who respond well to medication and have a good correlation between episodes of reflux in the pH monitoring and their symptoms, making them the best candidates for surgery.

Complication of paraesophageal hernias can be a twisting (volvulus) of the stomach with chronic gastrointestinal bleeding and resulting anemia. In extreme cases, incarceration may occur. In addition, the displacement of the stomach can lead to compression of the thoracic organs leading to respiratory problems.

Voluminous hiatal hernias may also remain asymptomatic or lead to the typical symptoms of GERD, early satiety, dysphagia, chest discomfort, or dyspnea. Severe acute symptoms are very uncommon.

A hiatal hernia is a common incidental finding on chest X-ray or CT scan. An erect chest X-ray can diagnose a hiatal hernia if it shows an air–fluid level behind the cardiac shadow, usually indicative of a paraesophageal hernia or voluminous hiatal hernia. An upper gastrointestinal barium study will provide diagnosis of a paraesophageal hernia or voluminous hiatal hernia in almost all cases. An axial hernia may be missed on chest X-ray, as it can spontaneously reduce, but can be confirmed by esophagogastroscopy with attention on the position of the gastroesophageal junction and length of esophagus.

Preoperative diagnosis always includes an endoscopy, an upper gastrointestinal barium study, and a manometry to exclude a functional cause. ACT scan is not absolutely necessary to plan the operation but can be helpful. An esophageal pH monitoring is done to confirm reflux and is recommended in the case of a voluminous hiatal hernia.

## Treatment

Treatment for hiatal hernias depends on both the type of hernia and the severity of the symptoms. For asymptomatic hiatal hernias, treatment is usually not necessary. For symptomatic hiatal hernias, treatment can be either conservative, such as medications for GERD, or operative.

Surgical repair of hiatal hernias can be challenging and is usually reserved for symptomatic patients, particularly in paraesophageal or voluminous hiatal hernias where there is an increased risk of complications with nonoperative management [10]. The surgical procedure aims to return the displaced organ to its normal position. Certain conditions including acute mechanical outlet obstruction, ischemia of the gastric wall, perforation, or severe bleeding demand an emergent repair.

Prior to minimal invasive techniques, hiatal hernias were repaired using an open transabdominal approach. Despite the fact that transabdominal open repair may still be appropriate in an emergency situation with peritoneal contamination or gastric necrosis, this approach is now widely replaced by laparoscopic surgery with reduced rates of perioperative morbidity and length of hospital stay [10], 12], 13].

### Robotic surgery vs. laparoscopic surgery

Laparoscopic surgery is associated with some technical limitations including an unstable video camera, 2D video system with lack of depth perception, straight instruments with limited range of motion, and poor ergonomics for the surgeon [14]. These limitations can be particularly challenging when operating on patients with voluminous hiatal hernias. Therefore, robotic surgery is becoming increasingly prevalent, since it overcomes most of the pitfalls of laparoscopic surgery and can be seen as a further advancement of the well-established, conventional laparoscopic technology. Robotic surgery provides a stable camera, 3D imaging, a 10-fold augmentation of the image, increased range of motion of robotic instruments, and improved ergonomics for the surgeon [15], [16], [17].

Robotic surgery affords excellent visualization that allows dissection high in the mediastinum and optimal hiatus reconstruction with low perioperative morbidity.

### Description of our surgical technique

We described our surgical technique in a recent book chapter about robotic upper gastrointestinal surgery [18]. Technical steps for primary hernia repair include hernia reduction, crural repair with nonabsorbable stitches with or without mesh, and fundoplication [19].

In our upper GI-unit, a robotic approach has been used in voluminous hiatal hernias for over 10 years using robotic surgery, currently using the da Vinci Xi robotic platform (Intuitive Surgical Inc., Sunnyvale, CA) whenever possible, according to the availability of the robotic system at the time of surgery but irrespective of the hernia size or any previous abdominal procedures. After completing the preoperative work-up, all patients who are scheduled for a hiatal hernia repair give their written consent to the procedure using a minimally invasive technique.

### Patient positioning and port placement

**Figure 1:**
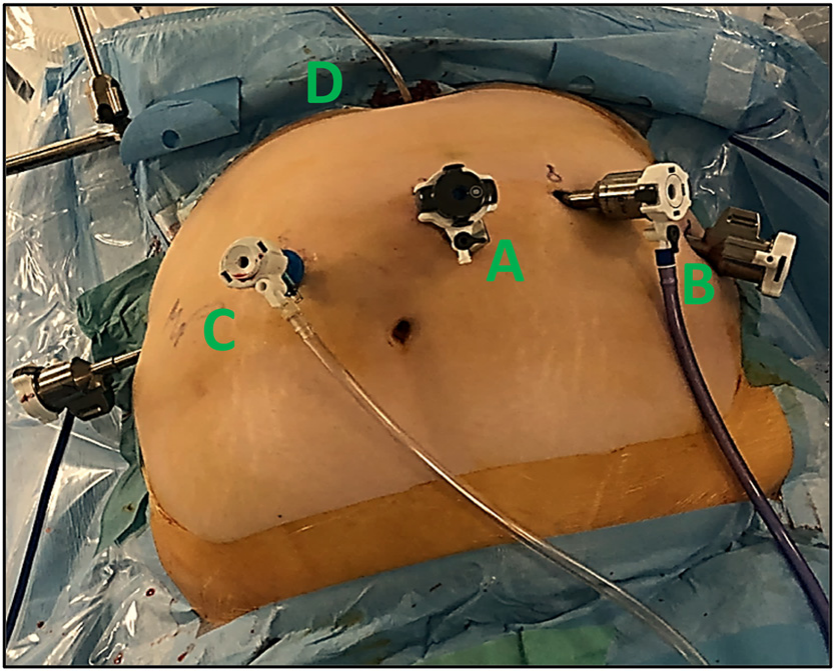
Trocar positioning. The first robotic trocar is for the camera and is placed 13 cm below the xyphoid process (A). Two robotic trocars are then placed on a horizontal line on the patient’s left side (B). Two trocars are placed on the right side, one additional robotic trocar and one laparoscopic trocar for the assistant (C). All trocars are placed at a distance of 8 cm from each other to avoid conflict. The Nathanson liver retractor is placed in the epigastric region (D).

For this surgery, patients are placed on the operating table in a supine position with leg straps, right arm adducted, left arm abducted, and in a reverse Trendelenburg position at 15°. For the robotic approach, five robotic trocars are placed. The first robotic 8 mm trocar (for the camera) should be placed at 13 cm below the xiphoid process, left para-umbilically. Two 8 mm robotic trocars should be placed on a horizontal line on the patient’s left side. Two trocars, one 5 mm laparoscopic, for the assistant, and one 8 mm robotic, should be placed on the patient’s right side. All trocars should have a space of 8 cm between them to avoid collisions between robotic arms during surgery. The da Vinci robot (Intuitive Surgical, Sunnyvale, CA, USA) is then docked on the right side of the patient (Figure 1).

**Figure 2:**
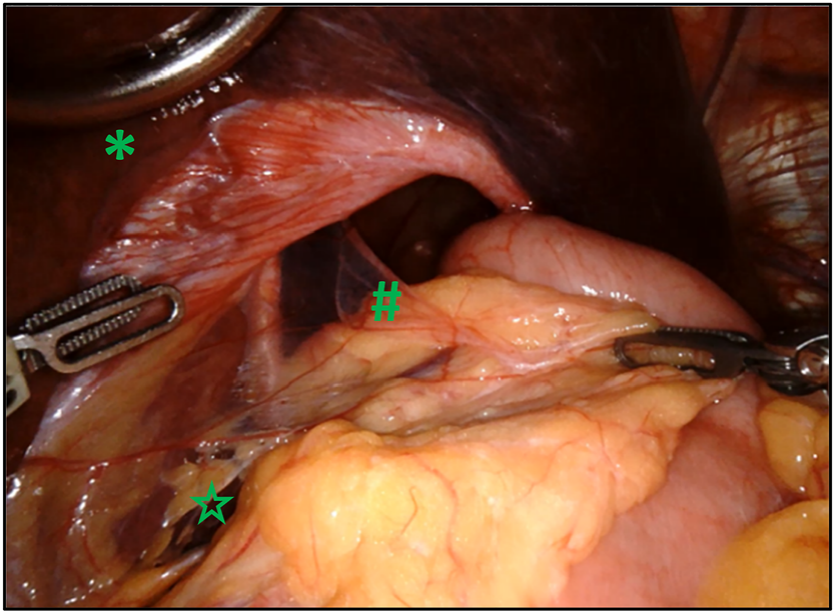
Voluminous hiatal hernia with a large hiatal defect (#). After introduction of the trocars into the abdominal space, the liver retractor (*) is placed in order to lift the left lateral lobe of the liver and give access to the esophageal hiatus. The procedure can then start with opening of the pars flaccida of the hepatogastric ligament (✩) to access the right diaphragmatic crus.

A Nathanson liver retractor is placed in the epigastric region to lift the left lateral segment of the liver to expose the hiatus. It should be noted that a standard nasogastric tube is initially placed to decompress the stomach during surgery, which is later replaced by a bougie dilator advanced into the stomach during esophageal mobilization (Figure 2).

**Figure 3:**
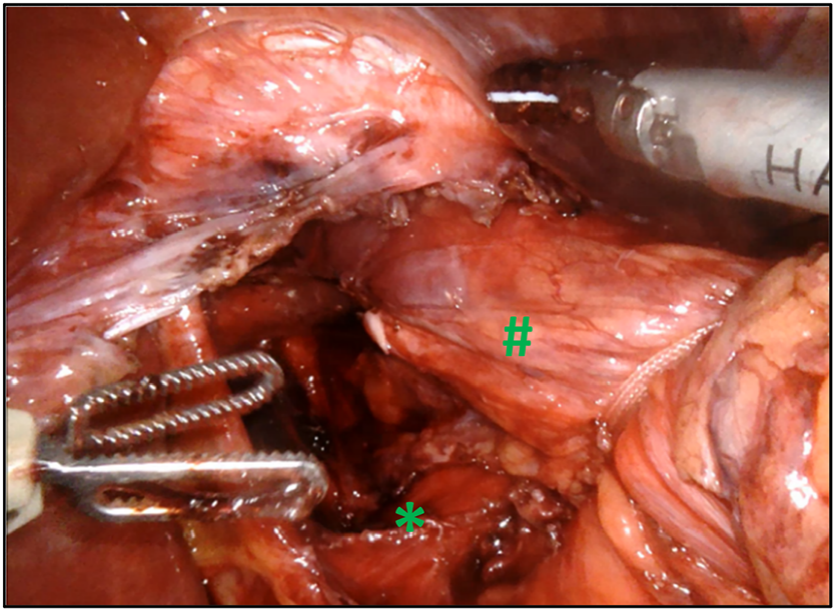
After dissection of the hernia sac and reduction of the hernia contents, the esophagus is mobilized in the posterior mediastinum in order to get a minimal intra-abdominal length of 3 cm (#). Next step of the procedure implies closing of the crural defect (*) using nonabsorbable interrupted sutures.

**Figure 4:**
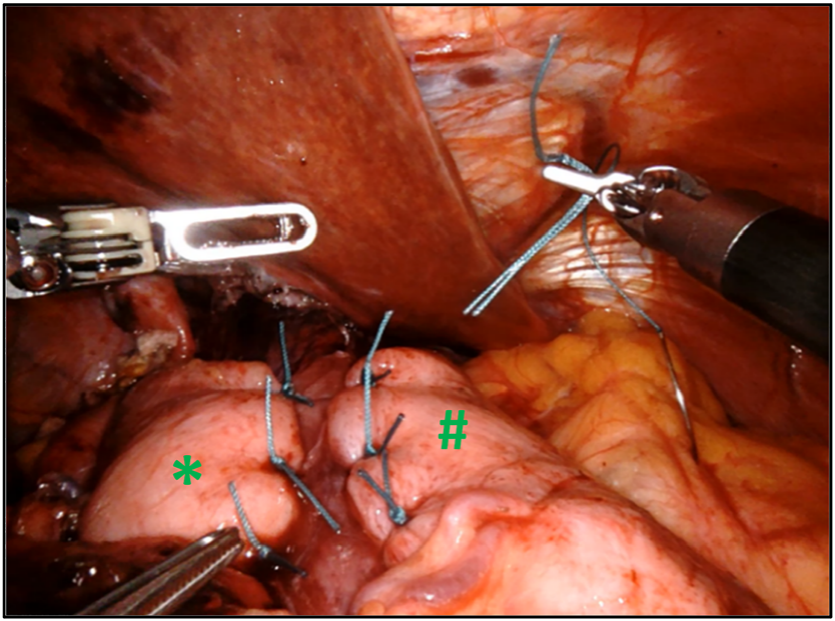
Toupet fundoplication with a 3–4 cm gastric wrap around 270° of the esophagus is obtained by fixing left (#) and right part (*) of the fundus to anterior muscular part of the esophagus with interrupted nonabsorbable sutures.

**Figure 5:**
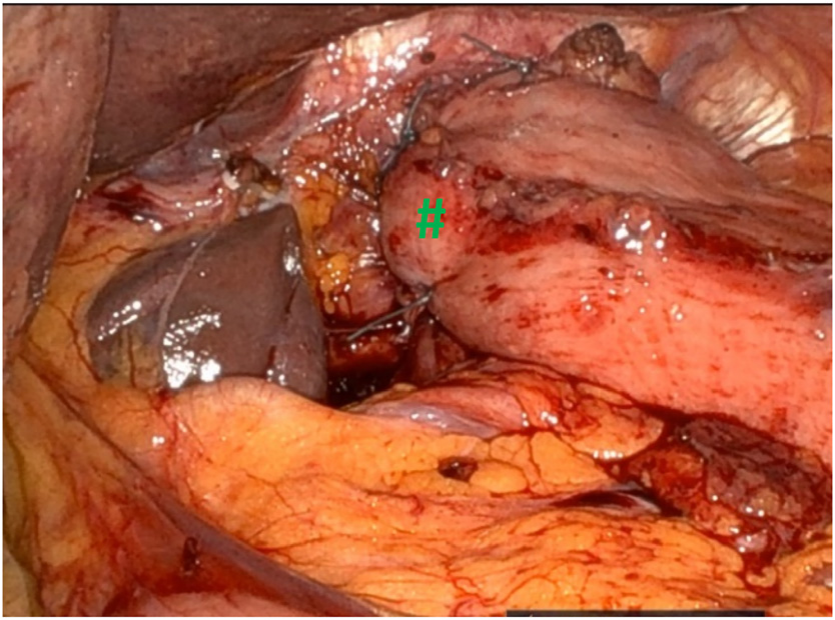
Dor fundoplication with a partial 180° wrapping of the gastric fundus (#) around the esophagus is performed as an alternative to Toupet fundoplication.

### Hernia reduction, sac excision, crural repair, and fundoplication

The procedure starts with the dissection of the hernia sac and the reduction of the hernia contents, after which the esophagus is mobilized in the posterior mediastinum (at least three cm) in order to achieve a correct repositioning of the gastroesophageal junction and an adequate intra-abdominal length. The hernia sac should be excised (Figure 3). A posterior cruroplasty is performed using three to five interrupted nonabsorbable sutures with PTFE pledgets. Mesh augmentation at the site of cruroplasty is not routinely used in our institution, but it can occasionally be considered for larger hiatal defects. However, we do not have a clear cutoff.

In all cases, a fundoplication is performed. Usually, we prefer to perform a posterior 270° fundoplication (Toupet) (Figures 4 and 5). When the patient is not known to have reflux, we can perform an anterior 180° fundoplication (Dor) as an alternative. At the end, all of the ports are removed with fascial closure of the 12-mm port site and we perform a chest x-ray to exclude a pneumothorax. Oral feeding is adapted after the operation with mixed food for 2 weeks.

## Discussion

The minimal invasive approach in the repair of hiatal hernias offers many benefits in comparison to the open approach such as reduced postoperative pain, faster recovery, and reduced risk of wound complications [12], 13]. Further, benefits of robotic hiatal hernia repair compared to traditional laparoscopic approach have been the subject of a number of recent publications. A summary of these publications is provided in Table 1.

**Table 1:** Reported series of hiatal hernia repair using robotic surgery.

				*Outcomes*			
*First authors*	*Year of publication*	*Data collection, years*	*n*	*Mean TOT, minutes*	*LOS, days*	*Conversion to open, %*	*30-day mortality, %*
Lin EL [20]	2022	2013–2017	169	197.4 ± 60.4	2.1 ± 3.6	n.c.	0 (0 %)
Tjeerdsma M [21]	2022	2014–2019	16	n.c	3 (2–5.8)	0 (0 %)	0 (0 %)
Benedix F^a^ [22]	2021	2016–2020	55	149.0 ± 4.1	3.6 (3.4–3.8)	1 (1.8 %)	0 (0 %)
Gerull WD [23]	2021	2009–2019	830	174.1 ± 63.8	1.8 ± 0.6	0 (0 %)	0 (0 %)
Hosein S [12]	2021	2015–2017	835	n.c	3.4 (5.0)	n.c	1 (0.1 %)
Ward MA [16]	2021	2010–2015	9,897	n.c	n.c	n.c	40 (0.4 %)
Arcerito M [24]	2020	2014–2018	70	223 (180–360)	1.6 (1–4)	0 (0 %)	0 (0 %)
O’Connor S [25]	2020	2012–2019	114	179	2.3	n.c	n.c
Soliman B [17]	2020	2012–2017	142	186.5 (152–232)	1.3 ± 1.8	1 (0.7 %)	0 (0 %)
Washington K [26]	2020	2013–2015	17	142 ± 25	3.4 ± 1.5	0 (0 %)	0 (0 %)
Mertens A [14]	2019	2011–2017	362	148 (128–174)	3 (3–5)	10 (2.8 %)	2 (0.6 %)
Vasudevan V [15]	2018	2011–2013	28	83.6 ± 24	2.8 ± 1.9	0 (0 %)	1 (3.4 %)
Niclauss N [19]	2017	2006–2016	82	212 ± 79.3	6.6 ± 3.9	3 (3.7 %)	0 (0 %)
Sarkaria I [27]	2017	2011–2014	24	277 (185–485)	4 (1–14)	0 (0 %)	0 (0 %)
Brenkman H [28]	2016	2011–2015	40	118 (62–173)	3 (1–15)	1 (2.5 %)	1 (2.5 %)
Galvani CA [29]	2016	2010–2015	61	186 (88–360)	2.6 (1–18)	0 (0 %)	0 (0 %)

TOT, total operative time in minutes; ^a^patients with GERD without hiatal hernias were included.

In 2016, Galvani and others showed that robotic surgery for the treatment of paraesophageal hernia was feasible and safe [29] as compared to results of laparoscopic approach detailed in a systematic review published 3 years earlier [30]. As corroborated in other studies, total operative time, the number of complications, and consequently the length of hospitalization tend to decrease with the experience of the surgeon and his team [20], 26]. These results were confirmed the same year in two other observational studies [27], 28]. Interestingly, Tolboom and others observed that results obtained with robotic surgery were comparable to conventional laparoscopic surgery for redo hiatal hernia and antireflux interventions, which are typically associated with higher morbidity and mortality [31]. In 2017, we published the results of our own experience with the robotic approach for hiatal hernia repair showing feasibility of a standardized technique with no major complications and low recurrence rate [19].

In 2018, Mertens and others published the results of a 7-year experience in a high volume center in robotic surgery for hiatal hernia repair [14]. They performed 211 primary surgeries and 151 redo surgeries and compared the results obtained for both types of operations. Results were not compared to conventional laparoscopic surgery in this single-arm, single-center retrospective study. However, it could show in this large patient cohort that robotic surgery could be adopted in giant or redo hiatal hernia repair at acceptable complication rates when compared to the available literature on the conventional laparoscopic approach. We should also mention two other observational studies with a small number of patients published at the same time, also showing acceptable complication rates compared to conventional laparoscopic surgery [15], 26].

Two studies with larger patient cohorts, both of them retrospective, showed that minimally invasive hiatal hernia repair, even in urgent/emergent settings, is superior to open hiatal hernia repair [12] and that improvements in perioperative outcomes can be observed in patients undergoing robotic-assisted approach [23]. In the study of Hosein and others, overall complications were significantly lower in the conventional laparoscopy group compared to robotic-assisted approach when all cases, including elective and emergent/urgent cases, were considered [12]. No other difference was observed, for example, hospital length of stay and costs were comparable between both groups. Interestingly, Gerull and others observed that total operative time was shorter for the robotic approach compared to laparoscopic, conversion rate to open was smaller, blood loss was lower, and hospital length of stay was shorter, even though more redo operations were included in the robotic approach group [23].

We can also mention other studies published in the recent years, of smaller size, however, showing comparable clinical results between laparoscopic and robotic surgery [17], 20]–22], 24], 25].

Nevertheless, Ward and others analyzed the Health Care Utilization Project National Inpatient Sample (HCUP-NIS) database in a study published in 2021 and found that robotic paraesophageal hiatal hernia repair was associated with significantly higher levels of complications compared to laparoscopic repair after performing a risk adjusted analysis (OR (95 % CI)=1.17 (1.07, 1.27), specifically regarding respiratory failure (OR (95 % CI)=1.68 (1.37, 2.05) and esophageal perforation (OR (95 % CI)=2.19 (1.42, 3.93) [16]. A subset analysis observed that this difference persists even in high volume centers (more than 20 operations per year). However, other perioperative outcomes, including hospital length of stay, were comparable.

As the publication by Ward and others includes the largest number of patients to date, it should be considered when assessing the relevance of using robotic surgery for hiatal hernias. However, it is important to note that this study is retrospective and utilizes a national-level database, which may introduce certain limitations in its analysis. For example, it did not conduct a subset analysis to compare outcomes based on the type of hiatal hernia.

Similarly, we need to underline that most of the publications cited here are observational or retrospective studies, with many of them comparing results between conventional laparoscopic surgery and robotic surgery at a single institution. In all cases, the studies are retrospective, even if the data were collected in a prospective manner.

In summary, robotic surgery appears to be safe and delivers results at least comparable to conventional laparoscopic surgery. Nevertheless, additional research, especially through randomized controlled trials, is necessary to confirm these encouraging preliminary findings.

At this point in the discussion, we would like to briefly address the issue concerning the cost of robotic surgery. Indeed, robotic surgery is typically associated with higher expenses compared to laparoscopic procedures, potentially imposing additional financial burdens on healthcare systems that are already under strain [32]. Nevertheless, the costs of robotic surgery are expected to decrease with more routine performance of robotic procedures and with improvements in surgical materials. This will hopefully lead to shorter operating times, reduced docking times, and higher rates of full robotic procedures [33]. In sum, cost–benefit ratios are anticipated to decrease in the coming years, further substantiating wider use of robotic surgery, including in cases of hiatal hernias [34], 35].

## Conclusions

The robotic approach for hiatal hernia repair has been gaining critical importance thanks to its significant technical advantages compared to the laparoscopic approach. Robotic surgery appears to be particularly well suited for the repair of voluminous hiatal hernias. In addition, the total operative time, complication rates, and hospital length of stay tend to decrease significantly over the years and depending on the experience of the surgeon.
